# A platform technology for generating subunit vaccines against diverse viral pathogens

**DOI:** 10.3389/fimmu.2022.963023

**Published:** 2022-08-18

**Authors:** Andrew Young, Ariel Isaacs, Connor A. P. Scott, Naphak Modhiran, Christopher L. D. McMillan, Stacey T. M. Cheung, Jennifer Barr, Glenn Marsh, Nazia Thakur, Dalan Bailey, Kenneth S. M. Li, Hayes K. H. Luk, Kin-Hang Kok, Susanna K. P. Lau, Patrick C. Y. Woo, Wakako Furuyama, Andrea Marzi, Paul R. Young, Keith J. Chappell, Daniel Watterson

**Affiliations:** ^1^ School of Chemistry and Molecular Biosciences, The University of Queensland, Brisbane, QLD, Australia; ^2^ The Australian Institute for Bioengineering and Nanotechnology, The University of Queensland, Brisbane, QLD, Australia; ^3^ CSIRO, Health and Biosecurity, Australian Centre for Disease Preparedness, Geelong, VIC, Australia; ^4^ The Pirbright Institute, Woking, United Kingdom; ^5^ Oxford Vaccine Group, Department of Paediatrics, Medical Sciences Division, University of Oxford, Oxford, United Kingdom; ^6^ Department of Microbiology, Li Ka Shing Faculty of Medicine, The University of Hong Kong, Hong Kong, Hong Kong SAR, China; ^7^ Laboratory of Virology, Division of Intramural Research, National Institute of Allergy and Infectious Diseases, National Institutes of Health, Hamilton, MT, United States; ^8^ Australian Infectious Disease Research Centre, The University of Queensland, Brisbane, QLD, Australia

**Keywords:** subunit, platform, viral, fusion, clamp, vaccine

## Abstract

The COVID-19 pandemic response has shown how vaccine platform technologies can be used to rapidly and effectively counteract a novel emerging infectious disease. The speed of development for mRNA and vector-based vaccines outpaced those of subunit vaccines, however, subunit vaccines can offer advantages in terms of safety and stability. Here we describe a subunit vaccine platform technology, the molecular clamp, in application to four viruses from divergent taxonomic families: Middle Eastern respiratory syndrome coronavirus (MERS-CoV), Ebola virus (EBOV), Lassa virus (LASV) and Nipah virus (NiV). The clamp streamlines subunit antigen production by both stabilising the immunologically important prefusion epitopes of trimeric viral fusion proteins while enabling purification without target-specific reagents by acting as an affinity tag. Conformations for each viral antigen were confirmed by monoclonal antibody binding, size exclusion chromatography and electron microscopy. Notably, all four antigens tested remained stable over four weeks of incubation at 40°C. Of the four vaccines tested, a neutralising immune response was stimulated by clamp stabilised MERS-CoV spike, EBOV glycoprotein and NiV fusion protein. Only the clamp stabilised LASV glycoprotein precursor failed to elicit virus neutralising antibodies. MERS-CoV and EBOV vaccine candidates were both tested in animal models and found to provide protection against viral challenge.

## 1 Introduction

Despite significant advances in medical research the frequency of outbreaks of emerging infectious diseases (EIDs) is increasing (1). Within the past two decades there have been viral epidemics from severe acute respiratory syndrome coronavirus (SARS-CoV), Zika virus, influenza viruses H5N1 and H1N1, Middle Eastern respiratory syndrome coronavirus (MERS-CoV), Ebola virus (EBOV), Lassa virus (LASV) and Nipah virus (NiV) (1, 2). Currently, SARS-CoV-2 is causing widespread mortality and global disruption and has exposed broad deficiencies in preparative and counteractive measures to control EIDs (3). Demographic and environmental factors suggest that the frequency of outbreaks will continue, with a growing global population (particularly aging demographics), increasing population densities, international movement of people and goods and continued encroachment on the habitats of animal reservoirs (2).

Vaccination is central to infectious disease control, but conventional developmental pipelines are ill-suited to react quickly to novel threats, requiring more than 10 years on average from discovery to licensure (4). The response to SARS-CoV-2 has challenged this paradigm with over 76 vaccine candidates announced within 3 months of the publication of its genome (5) and the first vaccines authorised for emergency use by the World Health Organisation (WHO) within 1 year (6). These efforts highlighted the utility of platform technologies that allow the exchange of specific viral antigens or genes into scaffold vectors to streamline the vaccine discovery phase. Such platforms can circumvent the need to physically transfer viral isolates from outbreak epicentres, allowing vaccine candidates and characterisation reagents to be synthesised remotely using genetic sequence information alone. Protein subunit, nucleic acid (DNA and RNA), replicating and non-replicating vectors, and virus-like particle vaccines all demonstrated pre-clinical efficacy for SARS-CoV-2 within months of genome publication, demonstrating the potential to rapidly generate vaccines across a variety of platforms (7–12).

These candidate vaccines largely focused on the ‘spike’ fusion protein as the target immunogen. For enveloped viruses, fusion proteins are often the primary targets for neutralising humoral immunity due to their surface presentation and critical functional role during host cell entry (13). Fusion proteins are dynamic molecular structures that assume distinct conformations before and after merging the viral and host membranes. The ‘prefusion’ conformation represents the metastable surface presentation on the infectious virion. Upon engagement, these surface antigens drive membrane fusion by irreversibly rearranging into a lower free-energy ‘postfusion’ conformation. Immunity elicited to prefusion-specific epitopes is often more protective than immunity to the postfusion form, and monoclonal antibodies (mAbs) with potent virus-neutralising activity that specifically bind prefusion epitopes have been well-described (14–20). Thus, stabilising the prefusion conformation is a common approach to enhance the efficacy of fusion protein vaccines (21).

Of the vaccine technologies mentioned above, subunit vaccines are particularly amenable to molecular characterisation and rational design (22). Recombinant viral fusion proteins can be modified to stabilise their conformation and then screened for the presentation of key protective and prefusion-specific epitopes. Cytotoxic and poorly immunogenic subdomains can be modified or omitted, and by only containing viral fragments without replicative components, the safety profiles of subunit vaccines are typically more predictable than other vaccine classes. Subunit vaccine production is scalable, and antigens can be designed and formulated for enhanced thermostability, reducing deployment costs to increase vaccine affordability and access (22). However, recombinant proteins can be poorly immunogenic and often require two or more doses with co-stimulatory adjuvants to elicit protective antibody and T-cell responses. Further, solubilising or otherwise modifying recombinant antigens can destabilise neutralising epitopes, requiring the introduction of stabilising mutations, multimeric scaffolds and/or heterologous motifs. Rational antigen design strategies have had success for clinically significant viruses such as RSV and HIV (14, 23) but these approaches are often dependent on detailed structural information, rendering them unsuitable for generic application and rapid mobilisation for novel EIDs.

Here we report a heterologous glycoprotein motif called the ‘molecular clamp’ which facilitates stabilisation and purification of viral fusion proteins. This technology has previously been described in a proof-of-concept production study using Achimota paramyxovirus and Wenzhou mammarenavirus (24), in application to a range of influenza hemagglutinin subtypes (25) and more recently, in a SARS-CoV-2 subunit vaccine that was effective in Phase 1 human clinical trials (9, 26). We build on those studies by presenting the streamlined generation of subunit vaccine candidates to four taxonomically diverse, WHO-listed priority pathogens: MERS-CoV (*Coronaviridae*), EBOV (*Filoviridae*), LASV (*Arenaviradae*) and NiV (*Paramyxoviridae*). We show that the clamped fusion antigens trimerise efficiently and present prefusion epitopes. We also show that vaccination with three of these antigens elicits neutralising immunity *in vitro* from BALB/c immunised sera. The vaccines also protect hamster and mouse models from viral challenge with mouse adapted-EBOV (MA-EBOV) and MA-MERS-CoV respectively. Importantly, it is shown that antigen of high purity can be obtained using the clamp as an immunoaffinity purification tag in a single step, enabling the generation of prefusion-stabilised subunit vaccine candidates without the use of target-specific reagents, demonstrating this technology’s potential for broader application and to novel emerging viruses.

## 2 Materials and methods

### 2.1 DNA vectors and protein expression

Amino acid sequences for NiV Malaysia strain fusion protein F (amino acids 1-483, GenBank ID: NP_112026.1), LASV Josiah strain glycoprotein precursor GPC (amino acids 1-427, GenBank ID: AAA46286.1), EBOV Mayinga 1976 strain glycoprotein GPΔmucin-like domain (MLD) (amino acids 1-303; 471-639, GenBank ID: AF086833.2) and MERS-CoV KFU-HKU-13 strain spike protein S (amino acids 1-1297, GenBank ID: AHX00711.1) were retrieved. The clamp domain was produced by connecting partial sequences encoding the gp41 subdomain of the HIV Env protein (amino acids 547-582, 625-662, GenBank ID: AAB50262.1) to the GP ectodomain, connected by a flexible linker (EBOV GPΔMLD: G2SG2; NiV F: GSG, MERS-CoV S: GSG; LASV GPC: G3SG3). Genetic constructs were codon optimised for expression in CHO-S cells (*Cricetulus griseus*) before nucleic acids were synthesised and cloned into a mammalian expression vector (pNBF; National Biologics Facility, Brisbane, Queensland, Australia) by inFusion cloning (ClonTech).

Plasmid DNA was extracted from bacterial cultures using PureYield DNA MidiPrep kit (Promega) and transfected into expiCHO cells using expiCHO expression kit (ThermoFisher). Suspension cultures were harvested 7 days post-transfection, before the supernatants were clarified by centrifugation at 5000 × g and then sterilised by filtration through 0.22 μm filters. Clamped proteins were purified from supernatant by immunoaffinity chromatography on a HiTrap NHS-activated column (GE Healthcare) conjugated with a molecular clamp-specific monoclonal antibody (mAb) (HIV1281; PDB 3P30) (17) using 400 mM NaCl PBS wash buffer and diethylamine elution buffer (5 mM EDTA, 100 mM Tris, 400 mM NaCl, 20 mM diethylamine, pH 11.5). Column elution fractions were neutralised with a 1:1 (v/v) ratio of 1 M Tris pH 6.8, concentrated, and buffer exchanged to PBS. Protein concentration was quantified using Nanodrop spectrophotometry.

Expression yield from CHO-S transient suspension culture was determined from 30 ml cultures performed in triplicate. Proteins were quantitated from absorbance spectra at 280 nm using a NanoDrop spectrophotometer (ThermoFisher), calibrated with extinction coefficients calculated from the full construct amino acid sequences using the Expasy ProtParam online tool (accessible from: https://web.expasy.org/protparam/).

Unstabilised MERS-CoV S was purchased from SinoBiological (CAT: 40069-V08B). The S protein originates from EMC/2012 strain (GenBank ID: AFS88936.1), consists of amino acids 1-1297 and is His-tagged. Control antigens for NiV (NiV FΔFP) and LASV (GPCysR4) have been previously described (18, 27).

### 2.2 *In vitro* protein characterisation

The purity and molecular weight of the proteins was assessed by loading 4 µg of denatured protein on a 4-12% SDS-PAGE (Biorad) under reducing conditions (100 mM dithiothreitol). Gels were stained in Coomassie brilliant blue R-250 for 1 hour and destained in 35% methanol and 10% acetic acid.

The oligomeric state of purified proteins was determined by size exclusion chromatography (SEC) using 50 µg of protein in a 300 μL loop connected to a Superose 6 Increase 10/300 GL (GE Healthcare Life Sciences) gel filtration column calibrated with a series of standards. Fractions of 1 mL were collected on the basis of elution volumes with peak absorbance values for subsequent analyses.

### 2.3 Antigen-specific monoclonal antibody binding

Protein antigenicity was assessed by enzyme-linked immunosorbent assay (ELISA) with prefusion-specific mAbs. For MERS-CoV S, receptor binding domain (RBD) mAbs 4C2 (28), m336 (29), LCA60 (30), MERS27 (31), MCA1 (32), JC57-14, CDC2-C2 (33) and D12 were used as well as a S2-specific mAb G4 (34). For LASV GPC, 37.7H (27), 12.1F and 25.10C (35) mAbs were analysed. For EBOV GP, h15758, h15765, h15959, h15960, h16042 (36), KZ52 (37), 2G4, 4G7, 1H3 (38), mab100, mab114 (39), and 13C6 (40) were used. For NiV F, prefusion-specific mAbs 5B3 (18) and mAb66 (41) were used. Briefly, 2 μg/mL of antigen in PBS was coated on a Nunc MaxiSorp 96-well plate (ThermoFisher) and incubated overnight at 4°C. Antigen was removed and plates were blocked with 150 μL per well of PBS with 0.05% Tween-20 supplemented with 5% milk diluent (blocking buffer) (KPL SeraCare) for 30 minutes at room temperature. mAbs were added to the plates pre-diluted in blocking buffer to 10 μg/mL and titrated 5-fold prior to incubation at 37°C for 1 hour. Plates were washed by water immersion thrice before addition of horseradish peroxidase (HRP)-conjugated secondary antibody (goat anti-human IgG) (ThermoFisher) diluted to 1:2000 in blocking buffer. Plates were incubated as before and washing was repeated. For signal generation, tetramethylbenzidine (TMB) (ThermoFisher) was added for 5-10 minutes at room temperature. Reaction was stopped with 1 M H_2_SO_4_ and optical density (OD) was measured at 450 nm. Background binding of mAbs against PBS was subtracted from binding to the respective antigen.

### 2.4 Transmission electron microscopy

SEC-purified antigens were deposited onto carbon-coated, glow-discharged 400 mesh copper grids (ProSciTech) at approximately 5 – 10 µg per ml. Samples were blotted off the grids and washed twice with water before staining with 1% (w/v) uranyl acetate for 2 mins. Grids were imaged using a Hitachi HT7700 Transmission Electron Microscope at 120 KeV and images were acquired using AXT 2kx2k CMOS. Subsequent micrograph processing was conducted using Relion 3.1 software, and contrast transfer functions of the images were corrected using CTFFIND. Particles were selected manually followed by reference-free alignment and two-dimensional classification.

Cryogenic electron microscopy (cryo-EM) was performed on NiV Fclamp proteins in complex with Fab fragments from 5B3, similar to a previous report (42). NiV Fclamp was mixed with purified Fab fragments at a w/w ratio of 1:2 respectively, and then incubated for 1 hour at 4°C. Samples were then loaded on to the Superose 6 Increase 10/300 GL column and size-exclusion was conducted as previously described. Fractions containing complexed antigens were identified by relative peak shifts and isolated, before being concentrated in 0.5 ml 3K MWCO concentrators. NiV Fclamp and 5B3 Fab complexes were diluted to 0.05 mg/ml and 4 µl was adsorbed onto flow discharged quantifoil grids (Q2/1). Grids were plunged frozen using EMGP2 system (Leica) and imaged on a Cryo-ARM 300 (JEOL) Field Emission Cryo-Electron Microscope equipped with a K3 detector (Gatan) in a super-resolution CDS acquisition mode. Movies were acquired with a 5 second exposure in super resolution mode at 0.1 s/frame using JADAS software at a magnification of ×50,000. This yielded a pixel size of 0.48 and a dose rate of 7.66 e/pix/sec. Data was motion corrected using MotionCor2 (v1.1.10), and contrast transfer functions (CTF) of each micrograph were determined using CTFFIND software. Any micrographs with aberrations were removed as informed by thon-ring images, and only micrographs with a resolution equal to or less than 4 Å were selected for downstream analyses. Initial 2D references were formed by manually picking particles in Relion 3.1 to inform auto-picking software with a box size of 640 pixels. 3D refinement was conducted using C3 symmetry with a total of 40,833 particles.

### 2.5 Animal immunisations and viral challenge studies

For the immunogenicity studies, seven-week-old BALB/c mice were housed in HEPA-filtered cages at the Australian Institute for Bioengineering and Nanotechnology (AIBN) Animal Facility at The University of Queensland, Australia. Procedures were approved by The University of Queensland Animal Ethics Committee (AEC numbers: SCMB/354/14/AIBN/UNIQUEST; SCMB/558/17). Each mouse was immunised under anesthesia by ketamine and xylazine with 5 µg of antigen or PBS adjuvanted with either 3 µg of Quil-A intradermally or 50 µg of Alhydrogel per dose intramuscularly as stated. Immunisations were conducted at 21-day intervals.

#### 2.5.1 EBOV challenge

All infectious work with MA-EBOV was performed in the high-containment laboratories at the Rocky Mountain Laboratories (RML), Division of Intramural Research, National Institute of Allergy and Infectious Diseases, National Institutes of Health. RML is an institution accredited by the Association for Assessment and Accreditation of Laboratory Animal Care International (AAALAC). All procedures followed standard operating procedures (SOPs) approved by the RML Institutional Biosafety Committee (IBC). Animal work was performed in strict accordance with the recommendations described in the Guide for the Care and Use of Laboratory Animals of the National Institute of Health, the Office of Animal Welfare and the Animal Welfare Act, United States Department of Agriculture. The study was approved by the RML Animal Care and Use Committee (ACUC). Procedures were conducted in animals anesthetized (inhalational isoflurane) by trained personnel under the supervision of veterinary staff. All efforts were made to ameliorate animal welfare and minimize animal suffering; food and water were available *ad libitum*.

For the EBOV challenge study, 4 - 6-week-old Syrian golden hamsters (*Mesocricetus auratus*) were immunised with either 5 µg of subunit antigen or PBS adjuvanted with 3 µg of Quil-A twice by intramuscular (IM) injection, 21 days apart. Positive control hamsters were immunised once with 100,000 PFU of VSV-EBOV at the 2^nd^ dose time point by the same route. Twenty-one days after the final immunisation, the hamsters were challenged with 1,000 LD_50_ (100 focus-forming units) of MA-EBOV by injection into the peritoneal cavity. Body weight changes were monitored daily until the hamsters recovered from the acute disease. Hamsters developing severe disease were euthanized following ACUC-approved endpoint criteria. Blood samples were collected by retro-orbital bleeding at the time of boost for subunit immunisations or the time of primary immunisation with VSV-EBOV (21 days pre-challenge) and 6 days prior to challenge. Terminal bleeds were collected *via* cardiac puncture at study endpoint (42 days post challenge (n = 6) or 4 days post challenge for tissue/blood sampling groups (n = 4)). At 4 days post MA-EBOV challenge, 4 hamsters per group were anesthetised and bled *via* cardiac puncture for virological analysis. Spleen and liver samples were collected and stored at -80°C. Virus loads were determined in hamster blood and tissue samples as previously described (43). Briefly, Vero E6 cells were seeded in 48-well plates the day before titration. Tissues were weighed, homogenised in 1 mL serum-free DMEM and tissue and blood samples were serially diluted 10-fold. Media was removed from cells and inoculated with each dilution in triplicate. After one hour, DMEM supplemented with 2% FBS, penicillin/streptomycin and L-glutamine was added and incubated at 37°C. Cells were monitored for cytopathic effect (CPE) and 50% tissue culture infectious dose (TCID_50_) was calculated for each sample (blood, per mL; tissue per mg) employing the Reed and Muench method (44).

#### 2.5.2 MERS-CoV challenge

The MERS-CoV mouse challenge experiment was approved by CULATR, HKU (CULATR 5067-19) and the Department of Health, the Government of the HKSAR under the Animals (Control of Experiments) Ordinance, Chapter 340 (19-384/385 in DH/SHS/8/2/3 Pt.32). The mouse study was carried out in strict compliance with animal welfare regulations. The mice were anesthetised by ketamine/xylazine when procedures were conducted. Standard guidelines prescribed in pain and distress in laboratory rodents and lagomorphs, Laboratory Animals 28, 97-112 (1994) were strictly followed and the well-being of animals were monitored daily with a scoring sheet to ensure minimal pain and distress experienced by the mice.

A congenic C57BL/6 mouse with mouse *DPP4* exons 10-12 replaced with the human *DPP4* codons was generated by Taconic Biosciences and provided by Paul McCray, University of Iowa to HKU (45). Human *DPP4* knockin mice, 6-8 weeks-old, were vaccinated with 1 µg or 5 µg of MERS-CoV Sclamp vaccine with MF59 adjuvant (Seqirus) or with 5 µg of MERS-CoV Sclamp without adjuvant. A comparator group of mice received 1.25 µg of formalin inactivated MERS-CoV and another group received PBS as a placebo. A booster vaccination of the same material was given after 3 weeks. Three weeks after the booster vaccination, mice were challenged with 10^4^ PFU of MA-MERS-CoV (clone 6.1.2) in 20 µl *via* intranasal route. Mice were weighed and monitored daily for 3 days. Five mice from each group were sacrificed on 3 days post infection with lungs harvested. Mice inoculated with MEM were included as mock infection group.

Lung tissues from infected mice were weighed and homogenised with TissueRuptor II (Qiagen, Hilden, Germany) in 1 ml PBS. Viral RNA was directly extracted from lung tissue homogenates of infected mice using RNeasy Mini Kit according to manufacturer’s instructions (Qiagen, Hilden, Germany). The RNA was eluted in 50 μl of RNase-free water and quantified. 100 ng of total RNA were used as template for cDNA synthesis by reverse transcription using SuperScript III kit (Invitrogen, San Diego, CA, USA). RT-qPCR was performed to determine the viral load of the infected mice lung tissues using LightCycler 480 SYBR Green I Master (Roche Diagnostics). Briefly, 5 µl of cDNA was used in each reaction with specific primers and probes targeting the nucleocapsid (N) gene of MERS-CoV as previously described (46). The relative abundance between different experimental groups was calculated by the ΔΔCt method normalised to mouse GAPDH.

To examine the histopathology, the lung tissues were fixed in 4% paraformaldehyde, embedded in paraffin, and stained with hematoxylin and eosin (H&E). Histopathological changes were observed using a Nikon 80i microscope and imaging system.

### 2.6 Measuring antigen-specific IgG responses

Serum reactivity to cognate antigens was determined by ELISA as described in section 2.3. In brief, ELISA plates were coated with 2 µg/ml of antigen and incubated overnight at 4°C. The plates were then treated with blocking buffer (1× KPL blocking solution concentrate (SeraCare) in PBST). Sera were serially diluted in blocking buffer before being added to the coated plate and incubated for 1 hour at 37°C. Bound antibodies were detected using goat anti- mouse IgG or goat anti-hamster IgG HRP conjugates (ThermoFisher) as appropriate. Bound conjugates were detected with 3,3′,5,5′-Tetramethylbenzidine (TMB) (ThermoFisher) before the reactions were quenched with 1 M H_2_SO_4_. Absorbances were read at 450 nm. Endpoint titres (EPTs) were then determined by taking the mean absorbance of the mock (serum-free) plus 3 standard deviations and interpolating this value into a one-site specific binding regression model using GraphPad Prism.

### 2.7 Virus and pseudovirus propagation and neutralisation assays

EBOV (Mayinga 1976, GenBank ID: AF086833.2), MA-EBOV (passage 3) (47) and VSV-EBOV (expressing EBOV-Kikwit GP (48)) were propagated in Vero E6 cells. The supernatants were clarified by centrifugation at 1500 × g for 10 min, aliquoted and stored in liquid nitrogen (MA-EBOV) or at -80°C (VSV-EBOV). MA-EBOV titers were determined by immuno-plaque assay. VSV-EBOV was titred on Vero E6 cells using standard plaque assay (49). EBOV (Mayinga 1976) neutralisation was quantified using a standard plaque assay in Vero E6 cell culture using previously described methods (50).

Live virus neutralisation assays were conducted under strict bio-containment procedures in the BSL4 laboratory at ACDP. Serial two-fold dilutions of sera were prepared in 96-well tissue culture plates in 50 µl DMEM (Dulbecco’s Modified Eagle’s Medium, supplemented with 1% antibiotic/antimycotic and 1% hepes). An equal volume of either NiV, MERS-CoV or LASV containing 200 TCID_50_ was added to each well and the virus-serum mix incubated for 45 min at 37°C in a humidified 5% CO_2_ incubator. Vero E6 cell suspension was prepared in DMEM containing 10% fetal bovine serum (FBS), then 100 µl of cell suspension containing 2 × 10^5^ cells/ml was added to each well and the plates incubated for three to five days. The wells were observed for signs of viral CPE and the titre was determined as the serum concentration in which viral CPE was not observed.

Pseudovirus neutralisation assays were conducted using lentivirus-based pseudotypes as previously described (51–53). Briefly, HEK293T cells were transfected with p8.91 (encoding for HIV-1 gag-pol), CSFLW (lentivirus backbone expressing a firefly luciferase reporter gene) and viral glycoprotein (NiV F + G or LASV GPC) using PEI transfection reagent. Supernatants containing pseudotyped virus were harvested at 48- and 72-hours post-transfection, pooled and centrifuged at 1,300 x *g* for 10 minutes at 4°C to remove cellular debris. Pseudo-particles were then titrated on target HEK293T cells to obtain a luciferase virus titre. For the micro neutralisation tests (mVNTs), sera were diluted in serum-free media in triplicate from a starting dilution of 1:10 and titrated 3-fold. A fixed titred volume of pseudo-particles was added at a dilution equivalent to 10^5^ - 10^6^ signal luciferase units in 50 µL DMEM-10% and incubated with sera for 1 hour at 37°C, 5% CO_2_. HEK293T cells were then added at a density of 2 ×10^4^ in 100 µL and incubated at 37°C, 5% CO_2_ for 48 hours. Firefly luciferase activity was then measured with BrightGlo luciferase reagent and a Glomax-Multi^+^ Detection System (Promega). Pseudotyped virus neutralisation titres were calculated by interpolating the point at which there was 50% reduction in luciferase activity, relative to untreated controls (neutralisation dose 50%, ND_50_).

### 2.8 Statistical analysis

Statistical analyses were performed using Prism 9 (GraphPad, San Diego, CA, USA). Yield differences were analysed with an unpaired, two-tailed t test. ELISA, virus and pseudovirus titres were analysed as log-transformations with ordinary one-way ANOVA and Tukey’s multiple comparisons test. MERS-CoV Ct values, eosinophil and histology scores were analysed with 2way ANOVA with Tukey’s multiple comparisons test. EBOV survival curves were analysed with a Mantel-Cox test. EBOV viral titres were analysed with ordinary one-way ANOVA with Tukey’s multiple comparisons test. Statistical significance is indicated as: p < 0.0001 (****), p < 0.001 (***), p < 0.01 (**), and p < 0.05 (*).

## 3 Results

### 3.1 Antigen design, expression and characterisation

**Figure 1:**
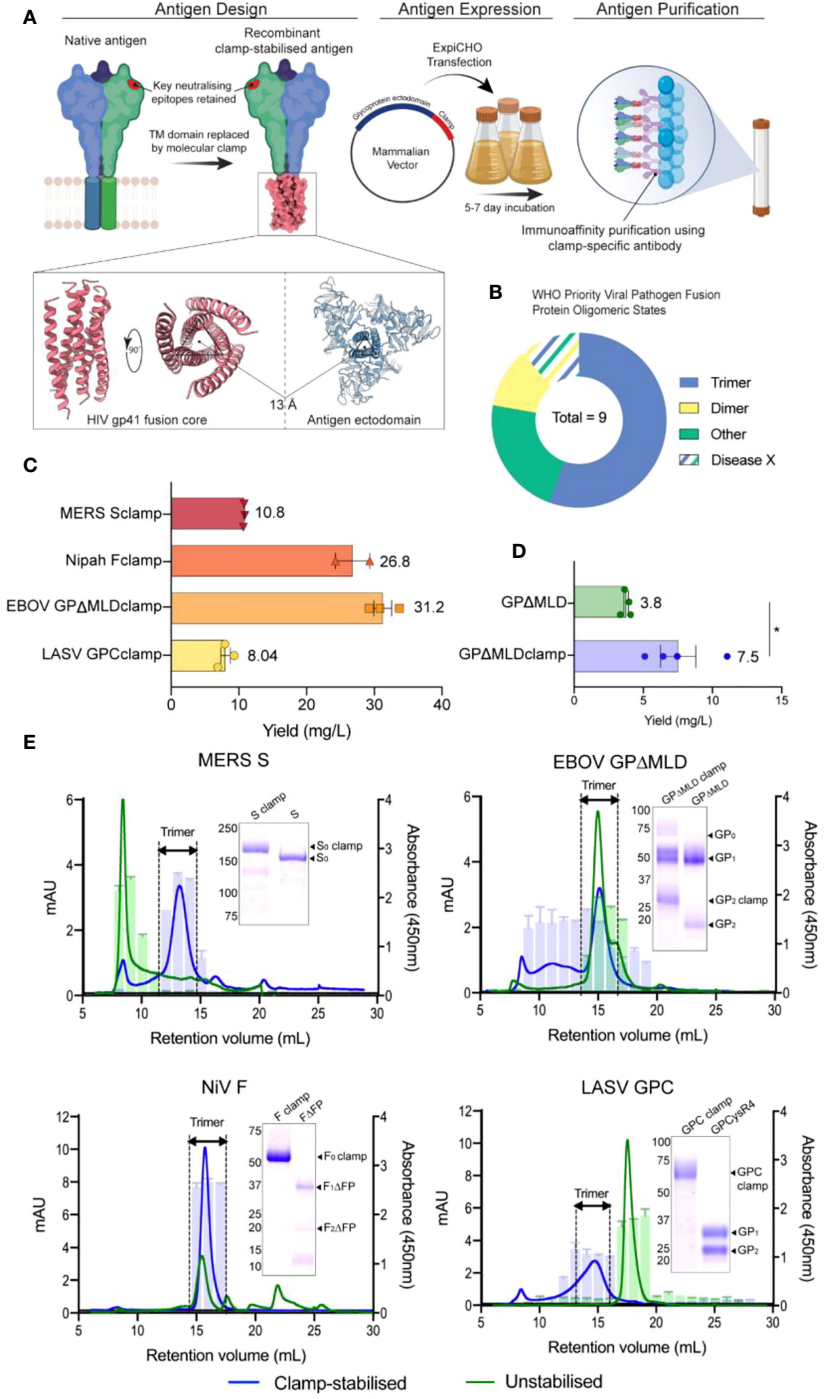
Design, generation and characterization of recombinant vaccine antigens. **(A)** The vaccine candidates were generated by genetically appending the clamp coding region in place of the native C-terminal transmembrane (TM) domain sequence. The constructs were cloned into a mammalian expression vector and expressed in ExpiCHO-S cells before the secreted antigens were purified using clamp-mediated immunoaffinity chromatography. **(B)** The WHO list’s priority viral pathogens of greatest significance for public health to focus R&D efforts towards. As the molecular clamp is a trimerization domain, the proportion of listed viruses containing a trimeric fusion protein that the clamp may be applied to are shown in blue. Disease X represents a yet unknown pathogen which may or may not also utilize a trimeric fusion protein. For the full list see (54). **(C)** The yield of the recombinant antigens from transient CHO-S suspension culture and clamp-mediated immunoaffinity chromatography. Error bars show SD from multiple expression runs. **(D)** Comparison of the yield recovered from anti-ectodomain affinity purification using EBOV mAb KZ52 of the clamp-stabilised and unstabilsed GPΔMLD constructs. The asterisk (*) indicates statistical significance determined by an unpaired, two-tailed t test (*p* = 0.0267). **(E)** The oligomerization states of the clamp-stabilised (blue) and unstabilised (green) antigens are presented as size exclusion chromatography UV traces (mAU) with prefusion specific monoclonal antibody reactivity presented as bar graphs (absorbance 450nm). Antibodies used were KZ52 for EBOV GP, 37.7H for LASV GPC, 5B3 for NiV F and 4C2 for MERS-CoV S. Coomassie blue-stained SDS-PAGE gels of the purified antigens are inserted.

The molecular clamp domain is derived from the HIV gp41 fusion core protein and consists of a pair of alpha-helices connected by a flexible linker which facilitates the formation of a hairpin tertiary structure (**Figure 1A**). These paired helix hairpins enhance trimerisation by coalescing with high affinity into thermostable, six-helix bundles *via* a series of intermolecular interdigitations (55). Of the 8 ‘known’ WHO-listed priority viral pathogens in need of vaccines and therapeutic interventions (one is a hypothetical ‘Disease X’), five contain trimeric fusion proteins that are potentially amenable to clamp-stabilisation (**Figure 1B**) (54).

Synthetic DNA fragments encoding NiV F, LASV GPC, MERS-CoV S or EBOV GPΔMLD ectodomains were cloned into mammalian expression vectors containing the molecular clamp sequence appended in place of their respective transmembrane (TM) domain coding regions. These produced constructs encoding NiV Fclamp, LASV GPCclamp, MERS-CoV Sclamp and EBOV GPΔMLDclamp. The recombinant antigens were transiently expressed in mammalian suspension cultures (Chinese hamster ovary, CHO-S) before purification using immunoaffinity with a clamp-specific monoclonal antibody (mAb) [HIV1281 (17)]. Yields ranged from 8 mg/L (LASV GPCclamp) to 31.2 mg/L (EBOV GPΔMLDclamp) for the clamped antigens (**Figure 1C**).

We attempted to generate unstabilised, ectodomain-only control antigens by similar methods. Purification using ectodomain-specific mAbs yielded EBOV GPΔMLD (purified with mAb KZ52 (37)), but control antigens were not recoverable for MERS-CoV S, NiV F or LASV GPC. Ultimately, to obtain unclamped control antigens for these viruses, best-in-class comparator proteins were used. These were LASV GPCysR4 (27), NiV FΔFP (18) and MERS-CoV S (MERS-CoV S1+S2 N-(AA1-1291)-His-C, SinoBiological, catalogue number: 40069-V08B). The yield of EBOV GPΔMLDclamp and GPΔMLD when purified with KZ52 showed incorporation of the clamp enhanced recoverable yield 2-fold for these antigens (p = 0.0267, **Figure 1D**). SDS-PAGE stained with Coomassie blue showed that clamp-mediated immunoaffinity chromatography recovered antigens of predicted molecular weight (MW) profiles and high purity (**Figure 1E**, inlayed).

Oligomerisation was assessed using size-exclusion fast protein liquid chromatography (SE-FPLC) with fractions assayed by ELISA using mAbs reactive to prefusion epitopes (**Figure 1E**). Sclamp (MERS-CoV) was predominantly trimeric (70% of total area under curve (AUC, mAU ml^-1^), 11.5-15 ml retention volumes (RV)) with some aggregation (9% AUC, 6-9.5 ml RV) and degradation/monomeric dissociation observed (13% AUC, 15.4-28 ml RV). The unstabilised counterpart, S, was largely aggregated (48% AUC; 6-10 ml RV) and exhibited a long ‘tail’-like profile (52% AUC; 10-30 ml RV), indicative of protein degradation. No local peak was observable corresponding to trimer within the S profile, and the receptor-binding domain (RBD)-specific mAb 4C2 (28) was unreactive to the S 11.5-15 ml RV fractions. However, 4C2 did bind to the stabilised Sclamp trimer and the S aggregated species.

EBOV GPΔMLDclamp produced high MW aggregates and the trimeric peak mAU maximum was reduced 2.13-fold relative to the unstabilised antigen (14.7 ml RV GPΔMLDclamp; 14.9 ml RV GPΔMLD) (**Figure 1E**). Trimer AUC comparisons between the EBOV constructs were not conducted as the GPΔMLD trimeric peak exhibited an overlapping, lower MW ‘shoulder’ consistent with monomeric dissociation (16.2 ml RV). However, aggregated protein accounted for the majority of the GPΔMLDclamp product (56% AUC, 0-13.5 ml RV) while only contributing 12% AUC to the total of GPΔMLD (0-13.5 ml RV).

NiV Fclamp exhibited a single major peak corresponding to trimeric oligomerisation (15.7 ml RV) and was reactive to mAb 5B3 (**Figure 1E**). The AUC of the FΔFP trimeric peak (40% AUC, 14-17 ml RV) was reduced 2.38-fold relative to Fclamp (94% AUC, 14-18.5 ml RV), with no mab 5B3 reactivity associated with the FΔFP trimer. Evidence of degradation and proteolysis was also observed in both SDS-PAGE and SE-FPLC profiles for the unstabilised FΔFP antigen.

Incorporation of the molecular clamp into GPCclamp (LASV) significantly enhanced trimerisation (64% AUC, 12.5-16.5 ml RV), with recombinant GPCysR4 presenting almost entirely as dissociated monomers (94% AUC, 16-19.5 ml RV). Both the monomeric GPCysR4 and the trimeric GPCclamp reacted with mAb 37.7H (**Figure 1E**).

Epitope presentation on the recombinant antigens was further examined by ELISA using broad panels of characterised mAbs targeted to key subdomains (**Supplementary Table S1**). The clamp-stabilised and control antigens typically bound the mAbs with comparable nanomolar apparent dissociation constants (kD). Notable discrepancies included MERS-CoV S, anti-’stem’ domain antibody, G4 (34), which demonstrated a 10-fold improvement of binding to the clamp-stabilised MERS-CoV antigen. NiV F prefusion-specific mAbs 5B3 and mAb66 bound Fclamp but were unreactive to FΔFP, consistent with previous reports that the unstabilised F antigen assumes a postfusion conformation (18). Epitope stability after storage in PBS at 4°C, 25°C or 40°C was assessed to examine the thermostability of the antigens in the absence of formulation stabilisers (**Figure S1**). For each clamped antigen it was found that the mAbs maintained low nanomolar affinities under each condition, including 4 weeks of storage at 40°C; the most stringent condition tested.

**Figure 2:**
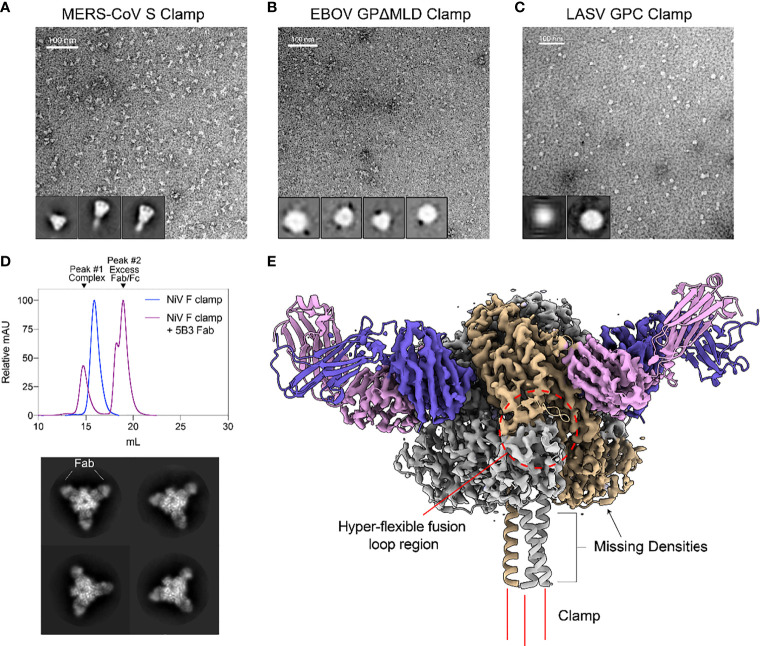
Structural validation and characterization of molecular clamped-stabilized antigens. **(A)** Representative negative stain TEM micrographs and 2D classes of MERS S Clamp, **(B)** EBOV GPΔMLD Clamp, and **(C)** LASV GPC Clamp. Clamp antigens were stained with 1% (w/v) uranyl acetate and imaged on a Hitachi HT7700 Transmission Electron Microscope at 120 kV. 2D classes were generated using Relion 3.1 software. **(D)** SEC of 5B3 Fab complexed or uncomplexed NiV F Clamp antigen separated on Superose 6 Increase 10/300GL column. Each data set is normalized to its maximal value. Below, representative 2D class averages of 5B3 complexed NiV F clamp. **(E)** 3.3 Å resolution cryo-EM structure of 5B3 complexed NiV F Clamp with the previously solved atomic structure (PDB 5EVM, PDB 6U1T) fitted in ribbon form. NiV F clamp is coloured in grey with a single monomer in gold. 5B3 heavy chains are coloured purple and light chains in pink. Unsolved clamp domain shown as three red lines at the C-terminus.

Negative stain transmission electron microscopy (TEM) was conducted to further investigate the oligomeric state and conformation for size-excluded trimer fractions of each clamped antigen (**Figure 2**). For MERS-CoV Sclamp, TEM showed a homogenous preparation of prefusion stabilised trimeric S protein. This was further confirmed using single particle analysis (SPA) on Relion 3.1 software which showed 2D class averages displaying S clamp trimer with a three-fold symmetry and a short-tail domain which indicated either the stem region of the spike or the molecular clamp (**Figure 2A**). Both micrographs and SPA of trimeric fractions of EBOV GPΔMLDclamp showed small, oblong and heart-shaped particles, indicative of the GP trimer (**Figure 2B**). Micrographs and 2D classifications of LASV GPC showed small spherical ~10 nm particles representative of the small globular LASV GPC trimer (**Figure 2C**).

As a proof of concept, we imaged NiV Fclamp using cryo-EM to further validate the authenticity of the prefusion structure. NiV Fclamp proteins were complexed with prefusion specific mAb 5B3 Fab fragments and imaged by cryo-EM. From the resulting micrographs, a total of 40,833 particles were used for SPA of the NiV F clamp 5B3 complexes. Using the gold-standard Relion 3.1 software pipeline, a cryo-EM map of NiV Fclamp 5B3 complex was generated with a final resolution of 3.3 Å. Here we observed a three-fold symmetry, showing three Fab fragments bound to a trimeric NiV F structure (**Figures 2D, E**). The previously solved atomic model of NiV F complexed with 5B3 (PDB 6TYS) (42) fit the cryo-EM map with a high degree of similarity. Of note, several regions of the NiV Fclamp antigen were not resolved. Densities corresponding to the stem domain of NiV F and the clamp domain were not observed in the reconstructions (**Figure 2E**). It is likely that the stem and clamp regions of the antigen are hyperflexible and therefore result in a high degree of movement, leading to a heterogenous sample and poor resolution around these regions. Furthermore, we observed a preferential top-down orientation of particles across cryo-EM micrographs, obscuring the stem and the clamp domains, potentially contributing to the loss of densities around these regions.

### 3.2 Vaccine immunogenicity and serum neutralisation in rodent models

**Figure 3:**
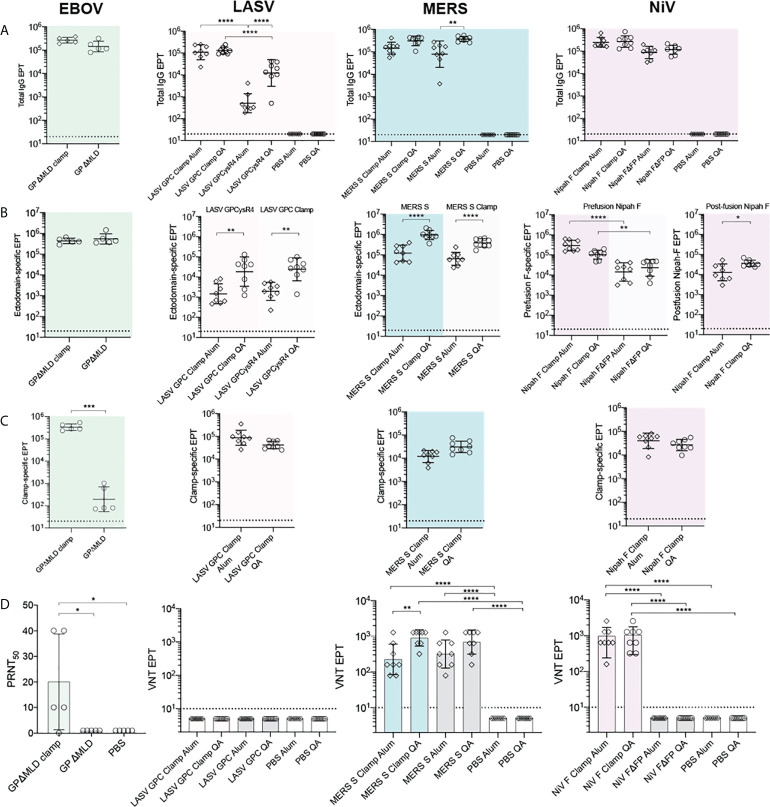
Immunogenicity and neutralization of virus. Serum reactivity in BALB/c immunised mice was assayed by ELISA and is presented as end-point titres (EPTs). **(A)** Sera groups were assayed against their cognate antigen (Total IgG), **(B)** an ectodomain only antigen (Ectodomain-specific) and **(C)** an influenza virus hemagglutinin-clamp antigen (Clamp-specific) (n = 5 for EBOV groups; n = 8 for LASV, MERS-CoV and NiV groups). **(D)** Neutralisation against live virus. Serum neutralization of EBOV was determined as the serum dilution factor required to neutralise 50% of the virus (PRNT_50_). For LASV, MERS-CoV and NiV, virus neutralisation titre (VNT) was determined as the inverse serum concentration at which CPE was not observed. *P* values were determined by one-way ANOVA using Tukey’s multiple comparison test. Statistical significance is indicated as: *p* < 0.0001 (****), *p* < 0.001 (***), *p* < 0.01 (**), and *p* < 0.05 (*). Error bars show SD.

We next determined the antigen-specific reactivity of sera elicited to the recombinant vaccine candidates. For the NiV, LASV and MERS-CoV vaccines, BALB/c mice (n = 8) were immunised with 5 µg of recombinant antigen intradermally (ID), adjuvanted with 3 µg of Quil-A^®^ (QA; Brenntag Biosector), or intramuscularly (IM), adjuvanted with 50 µg of Alhydrogel (Alum; Croda), three times at 21-day intervals. BALB/c mice (n = 5) were immunised with the EBOV antigens with the same dose and regimen, however only QA was tested as an adjuvant in these groups. All vaccinations were well tolerated. Twenty-one days after the final dose, blood was collected by cardiac puncture and sera reactivities to the cognate immunogens were assayed by ELISA (**Figure 3A**). The clamp-stabilised and unstabilised control antigens elicited broadly consistent IgG titres between immunogen pairs except in the case of LASV GPCclamp, which induced a significantly higher IgG titre than GPCysR4 under both adjuvant conditions (QA, p = <0.0001; Alum, p = <0.0001) (**Figure 3A**). There was a trend of QA-adjuvanted antigens eliciting higher IgG titres than Alum-adjuvanted equivalents, but this only reached statistical significance for LASV GPCysR4 (p = <0.0001) and MERS-CoV S (p = 0.004).

To differentiate fusion protein ectodomain reactivity from off-target immunity elicited to the molecular clamp, immunised serum was assayed against a clamp-stabilised influenza virus H3clamp antigen (**Figure 3C**). Similarly, the respective ‘unclamped’ control antigens were assayed to determine viral ectodomain IgG titres for MERS-CoV, LASV and EBOV (**Figure 3B**). For NiV, both foldon stabilised F and the FΔFP control (18) were used to determine prefusion and postfusion ectodomain-specific reactivity, respectively. This analysis indicated that the clamp is immunogenic, but the majority of the IgG titres were elicited to viral ectodomains in all cases except for LASV GPCclamp (clamp reactivity: 96.92%, Alum-adjuvanted; 61.45%, QA-adjuvanted). For EBOV, NiV and MERS-CoV clamped antigens, clamp-specific reactivity ranged from 3.8% - 21.5%.

The neutralisation potency of the immunised sera was then determined using viral isolates in BSL4 biocontainment facilities (**Figure 3D**) and pseudoviruses expressing wild-type viral surface antigens for LASV and NiV (**Figure S2**). Immunisation with adjuvant-matched, clamped and control MERS-CoV antigens induced comparably potent virus neutralising responses relative to PBS controls (Sclamp, QA and Alum, p = <0.0001; S, QA and Alum, p = <0.0001), with QA inducing neutralisation to a greater degree than Alum for Sclamp (p = 0.0024). For NiV, immunisation with clamp-stabilised F elicited a significantly enhanced neutralising response relative to FΔFP (QA and Alum, p = <0.0001), consistent with reports that the majority of neutralising epitopes are presented on the prefusion antigen conformation (41, 42, 56). Stabilised EBOV GPΔMLDclamp elicited significantly higher levels of neutralising antibodies (nAbs) in comparison to the GPΔMLD control. For LASV, neither GPCclamp nor GPCysR4 were able to induce a significant neutralising response in both the viral and pseudoviral systems irrespective of the adjuvant used.

### 3.3 Viral challenge models

For MERS-CoV and EBOV, the ability of the vaccine candidates to protect against lethal viral challenge was examined in well-characterised rodent models. The MERS-CoV study used *hDPP4* knock-in C57BL/6 mice (n = 5) (45), immunised twice, three weeks apart, with 1 or 5 µg of MERS-CoV Sclamp and 25 µl of squalene adjuvant MF59 (Seqirus). Infected and uninfected PBS placebo controls and an unadjuvanted 5 µg Sclamp cohort were also included. Finally, a formalin-inactivated MERS-CoV (1.25 µg) positive control was added, which has been shown to enhance lung disease in this model (45).

**Figure 4:**
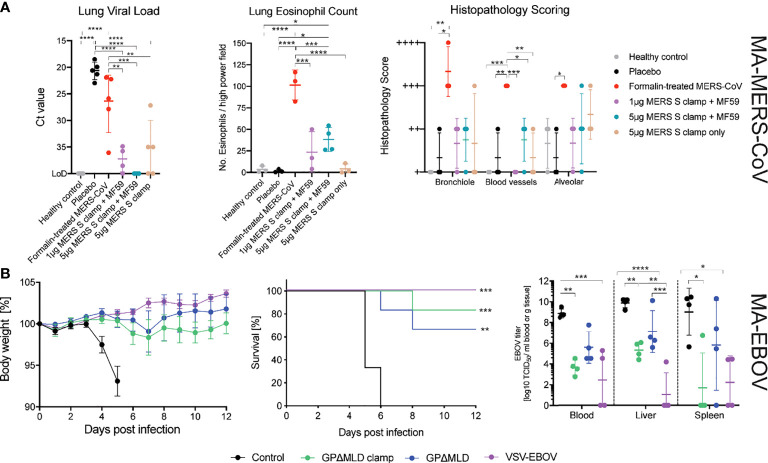
MERS-CoV and EBOV challenge models. **(A)** Congenic C57BL/6 DPP4 knock-in mice were given prime and boost immunisations before challenge with 10^4^ PFU of MA-MERS. The tissues were then assessed for infiltration of the virus and histopathology. Asterisks indicate statistical significance calculated by a two-way ANOVA with Tukey’s multiple comparisons test. **(B)** Syrian golden hamsters were immunised with clamped influenza virus H3 hemagluttinin (control), clamp-stabilised and unstabilised recombinant GPΔMLD or VSV-EBOV and challenged with 1,000 LD_50_ of MA-EBOV (n = 10). Body weights and survival of the hamsters was monitored following the challenge (n = 6). Four days post-challenge, hamsters were euthanized for virological analysis of the blood and tissues (n = 4). Statistics are calculated using a two-way ANOVA grouped analysis with Tukey’s multiple comparisons test. Survival curves were analysed with a Mantel-Cox test. Statistical significance is indicated as: *p* < 0.0001 (****), *p* < 0.001 (***), *p* < 0.01 (**), and *p* < 0.05 (*).

Three weeks after the final dose, mice were intranasally challenged with 1 x 10^4^ pfu of MA-MERS-CoV. Three days post-challenge at the peak of infection the mice were sacrificed, and the lungs were assessed histologically and assayed for viral load (**Figure 4A**). Viral RNA in the lungs of mice immunised with 5 µg MERS-CoV Sclamp + MF59 was below the limit of detection (LOD = 40 mean Ct). This constituted a ≈65,000-fold reduction in viral load relative to 1 µg MERS-CoV Sclamp + MF59 (mean Ct = 37), and a ≈500,000-fold reduction relative to the placebo control (mean Ct = 21).

Disease severity was assessed by histopathological staining for eosinophil infiltration and scoring of lung pathology (**Figure 4A**). Mice were also assessed for weight loss on days 1-3 post infection however, no significant change in weight was observed for any of the groups (data not shown). The formalin inactivated MERS-CoV induced enhanced eosinophil infiltration into the lungs and increased the percentage of lung tissue affected. Eosinophil infiltration and the percentage of lung tissue affected were higher in MERS-CoV Sclamp/MF59 vaccinated animals compared to placebo. However, both measures of lung inflammation for Sclamp immunised mice were consistently lower than animals vaccinated with the formalin inactivated virus. For the 1 µg and 5 µg MERS-CoV Sclamp + MF59 the average number of eosinophils per high-power viewing field at 400× magnification in lung sections stained with Congo Red (Sigma-Aldrich) was 14 and 27, respectively, compared to 90 for the formalin treated virus. Additionally, the percentage of lung tissue affected was consistently lower for mice vaccinated with MERS-CoV Sclamp at either dose + MF59 compared to formalin treated virus.

We then determined the protective efficacy of vaccination with the recombinant EBOV subunit antigens in a lethal EBOV challenge model. Syrian golden hamsters were immunised twice at a 21-day interval with 5 µg of EBOV GPΔMLDclamp or GPΔMLD, adjuvanted with 3 µg of QA. Negative control animals were immunised with 5 µg of QA-adjuvanted influenza virus H3clamp, and positive control animals were immunised once with 100,000 PFU of VSV-EBOV (48, 57). Twenty-one days after the final immunisation, all hamsters were challenged with 1000 LD_50_ of MA-EBOV (**Figure 4B**).

Both the GPΔMLDclamp and GPΔMLD subunit vaccines were protective, with 5 of 6 and 4 of 6 hamsters surviving, respectively. Body weights remained stable throughout the observation window; never fluctuating by more than 10% for all groups except for the H3clamp control (**Figure 4B**). Virological analysis of the blood and tissues 4 days post-infection (n = 4) showed a significant reduction of viral titres from animals immunised with GPΔMLDclamp and VSV-EBOV relative to the control group (**Figure 4B**, viral titre). Viral clearance associated with GPΔMLD vaccination was not significant. Contrasting titres across all samples for animals immunised with either GPΔMLDclamp or VSV-EBOV revealed insignificant differences. Mean viral titres in the liver and spleen were approximately 100-fold higher for animals immunised with GPΔMLDclamp than were observed in the VSV-EBOV cohort, although titres in the blood were 5-fold lower by this comparison. In the spleen, the disparity was attributable to one hamster in the GPΔMLDclamp group, with the remaining three fully clearing the virus from this organ. Three of 4 hamsters from the VSV-EBOV group showed no signs of virus in the liver, while all other animals presented substantial titres.

## 4 Discussion

The response to the SARS-CoV-2 pandemic has shown that with current technologies and sufficient resources, the time required to progress from vaccine design to human trials can be narrowed from years to months. Multiple vaccine modalities have produced effective candidates while offering different strengths and limitations, and future EID outbreak risk may be best mitigated by having vaccine development pathways established across a variety of platforms. Previously, we have shown the application of the molecular clamp in a SARS-CoV-2 spike subunit vaccine and demonstrated safety and efficacy in preclinical (9) and early clinical trials (26). While that study was ultimately discontinued due to interference with certain HIV diagnostics (58), it demonstrated that this platform technology can be rapidly and effectively mobilised in a pandemic. To resolve this issue, work is underway to explore modifying the current clamp and investigating the use of alternate motifs.

Using the clamp domain as an affinity tag, we showed that pure yields of the subunit antigens can be recovered without target-specific reagents (**Figures 1C–E**). Purification of recombinant proteins by immunoaffinity chromatography typically requires the introduction of affinity tags [such as C-myc (59) or FLAG tags (60)] or using antibodies specific to the antigen itself. The latter approach requires screening infected individuals or immunising animal models with the antigen of interest and identifying monoclonal antibodies that are amenable to antigen purification (61). This process can take months, extending the time required to generate subunit antigens in an emergency setting. However, the clamp domain’s affinity tag function eliminates this step. The yields of the stabilised antigens from small-scale transient mammalian culture ranged from 8 - 31.2 mg L^-1^ (**Figure 1C**). We have previously demonstrated that the level of antigen production can be improved by roughly 50-fold by transitioning to stable clonal cell lines in industry-scale bioreactors. In that instance, upscaling production also improved product homogeneity (9). It is anticipated that clamp-mediated, industry-scale production is also compatible with the vaccine candidates described here.

The production of unclamped but otherwise equivalent control antigens was only successful for EBOV GPΔMLD, suggesting that in the absence of other stabilising modifications the clamp was necessary for expression of the MERS-CoV, NiV and LASV antigens. This was despite validation of anti-ectodomain purification using the clamped counterpart of each control antigen (EBOV, **Figure 1D**; MERS-CoV, NiV and LASV, data not shown). For EBOV, where direct clamped and unclamped comparisons were possible, antigen yield recoverable by anti-ectodomain immunoaffinity purification was roughly doubled when the clamp was present (**Figure 1D**). Importantly, including the clamp domain enhanced trimerisation for MERS-CoV, NiV and LASV (**Figure 1E**) to better reflect the native oligomeric state of the fusion antigens. For EBOV GPΔMLDclamp, aggregation was observed that reduced the overall proportion of trimer to below the corresponding control. However, while a greater proportion of the EBOV control antigen was trimeric, it also exhibited evidence of dissociating into monomers that was absent from GPΔMLDclamp. Aggregation is a common issue in recombinant protein expression and can occur for a variety of reasons including culture conditions, protein yield and intrinsic antigen instability (62). To circumvent the presence of aggregate for GPΔMLDclamp, an additional purification step can be introduced to isolate the trimeric fractions by gel filtration, for example. This is routinely performed to ensure the homogeneity of recombinant proteins and is a scalable process (62).

Remarkably, each of the four clamp-stabilised vaccine candidates were found to be stable at 40°C for at least 4 weeks in PBS without formulation stabilisers. (**Figure S1**). This suggests that optimised formulations of these vaccines have potential to be distributed without stringent temperature controls. Preserving the safety and efficacy of vaccine doses by maintaining unbroken cold chains during vaccine deployment can account for up to 80% of costs (63), so by reducing or eliminating dependence on cold chain distribution clamp-stabilised vaccines could significantly improve availability for at-risk populations. The impact of vaccine thermostability should not be understated, with vaccine access having been described as “the greatest challenge for protection of the human population against serious infectious disease” (64).

Robust murine IgG responses were elicited to the LASV, MERS-CoV, NiV and EBOV clamped subunit vaccines (**Figure 3**). This corresponded to significant viral neutralisation *in vitro* for three of the four vaccine candidates (MERS-CoV Sclamp, EBOV GPΔMLDclamp and NiV Fclamp). However, neutralisation was not observed for LASV GPCclamp immunised sera despite the antigen being shown to bind known nAbs 37.7H, 25.10C and 12.1F (**Table S1**). Notably, LASV GPCclamp was unprocessed at the internal GPC1-GPC2 cleavage site (**Figure 1E**, inlayed), which has been described as necessary for GPC to properly adopt its prefusion conformation and could affect immunogenicity despite the observed nAb reactivity (65). Another contributing factor may be putative shielding of the GPC protein surface with extensive glycosylation (66). GPC shielding is consistent with the observation that LASV GPCclamp generated higher IgG titres to the clamp domain relative to the ectodomain, despite the clamp accounting for approximately 1/10th of the total antigen by molecular weight. Therapeutic antibodies and convalescent sera have shown variable efficacy for treating LASV disease which may indicate why the generation of protective humoral immunity from a LASV vaccine remains elusive despite considerable efforts (67). Cai *et al.* (2020) (68) showed that immunisation with a codon deoptimised, whole virus LASV vaccine conferred full protection in guinea pigs challenged with guinea pig-adapted LASV while only 1 of 16 animals produced detectable nAbs. In the LASV case, protection appears to be conferred by alternate immune mechanisms such as antibody-dependent cellular cytotoxicity (ADCC) (69). EBOV also shrouds epitopes on GP with glycans but is sensitive to nAbs at key immunogenic sites (36, 70, 71). For EBOV, total IgG titres have been proposed as a stronger correlate of protection than *in vitro* neutralisation or ADCC (72), which may indicate why protection from lethal viral challenge was observed (**Figure 4B**) despite weak virus neutralisation in plaque assays. Taken together, these results indicate that it may be worth persisting with this candidate LASV vaccine despite the absence of *in vitro* neutralisation.

While the lack of replicating elements and the absence of off-target pathogen associated molecular patterns confers greater safety profiles to subunit vaccines, adjuvants are typically necessary to achieve protective B and T cell responses (22). The LASV and MERS-CoV immunised sera showed significant enhancement of ectodomain-specific IgG titres for QA-adjuvanted doses relative to Alum for both control and clamped antigens, indicating that immunogenicity could be further augmented by exploring different adjuvants (**Figure 3B**). Ideally, a vaccine will induce both humoral and cellular immunity to generate stronger protection and exploit the differing susceptibilities of the viral targets (LASV, for example). While only IgG induction and neutralisation were assessed here, the previously mentioned SARS-CoV-2 Sclamp study showed that, in addition to robust humoral immunity, strong CD8 T-cell responses were elicited in mice when immunised with SARS-CoV-2 Sclamp adjuvanted with MF59 (9). However, previous research comparing a variety of adjuvants with different subunit vaccines has shown that the relationship between antigen, adjuvant and the induction of specific immunity can be unpredictable (73). This was illustrated by NiV Fclamp showing a trend of Alum enhancing ectodomain-specific immunity over QA, contrary to the observations for MERS-CoV and LASV (**Figure 3B**). Future studies should assess the effects of different adjuvants on the induction of alternate immune pathways with these vaccines.

The recent resounding success of mRNA vaccines for mitigating the impact of COVID-19 has launched mRNA vaccines into the wider consciousness, with many speculating that they may represent the future of vaccinology (74). The major advantages of the platform have been efficacy and the ability to generate vaccines with unprecedented speed (5, 8, 11). The current limitations of mRNA vaccines are their formulation stability and associated dependency on ultracold storage and transport (75) and uncertainty about long-term safety and immunological memory due to the nascence of the technology. Each of these limitations may yet be surmounted with continued investigation and development of the technology and its delivery vehicles. However, fusion antigens translated *in vivo* from mRNA vaccines still require that the correct antigen conformation be presented to the immune system. For example, both the Pfizer BNT162b2 and the Moderna mRNA-1273 vaccines contain codon substitutions that introduce prolines to constrain the prefusion conformation of the expressed vaccine antigen (76, 77). In both cases, the necessary structural understanding to rationally target the K986P and V987P substitutions was greatly accelerated by adapting established insights from prior structural investigation of the MERS-CoV spike protein (16). Assessing the application of the molecular clamp to mRNA vaccines presents another interesting line of enquiry and may offer a generic stabilisation strategy for *in vivo* mRNA expression of novel EID fusion antigens without prior characterisation of analogous proteins.

Here we have demonstrated the application of the molecular clamp platform technology to generate subunit vaccines for viruses from four divergent families. Conventional development of subunit vaccines can take longer than other classes of vaccine, offsetting the benefits of an established safety record and capacity for economical production and deployment. By exploiting the clamp domain’s dual functions of stabilising immunologically important epitopes on trimeric fusion proteins while acting as an affinity tag, the subunit vaccine discovery phase can be shortened substantially, bringing development times in line with those of other platforms and offering an additional tool for emergency response.

## Data availability statement

The raw data supporting the conclusions of this article will be made available by the authors, without undue reservation.

## Ethics statement

The animal study was reviewed and approved by The University of Queensland Animal Ethics Committee, RML Animal Care and Use Committee (ACUC) and the Committee on the Use of Live Animals in Teaching and Research (CULATR), HKU.

## Author contributions

Conceptualisation and invention of the clamp technology by PY, KC and DW. Clamped vaccine candidate and EBOV control antigen preparation, characterisation, immunisation and serum EPT studies by AY, AI, CS and CM. Additional animal handling and immunisation by SC. Electron microscopy by NM, AI and CS. Live virus neutralisation assays by JB and GM. MERS-CoV challenge studies by KL, HL, K-HK, SL and PW. EBOV challenge studies by WF and AM. Pseudovirus assays and analysis by AI, NT and DB. Writing-original draft AY. Writing-review and editing AI, CS, DW and KC. All authors contributed to the article and approved the submitted version.

## Funding

This research was funded by NHMRC Project grant APP1144025 and NHMRC Development grant APP1125107. This study was also funded in part by the Intramural Research Program, NIAID, NIH. NT and DB were funded by The Pirbright Institute’s BBSRC institute strategic programme grant BBS/E/I/00007031, with NT receiving studentship support from BB/T008784/1.

## Acknowledgments

Purified, recombinant NiV sF glycoprotein in its postfusion conformation (NiV FΔFP) was prepared and provided by Lianying Yan and Christopher Broder, Uniformed Services University, Bethesda, Maryland, USA. Recombinant LASV GPCysR4 control antigen was provided by Dr. Erica Ollmann Saphire, Center for Infectious Disease and Vaccine Research, La Jolla Institute for Immunology, La Jolla, CA, USA. MF59 adjuvant was provided by Seqirus Inc. Holly Springs, NC 27540 USA. **Figure 1A** was created using BioRender.com.

## Conflict of interest

The authors declare that the research was conducted in the absence of any commercial or financial relationships that could be construed as a potential conflict of interest.

## Publisher’s note

All claims expressed in this article are solely those of the authors and do not necessarily represent those of their affiliated organizations, or those of the publisher, the editors and the reviewers. Any product that may be evaluated in this article, or claim that may be made by its manufacturer, is not guaranteed or endorsed by the publisher.

## References

[1] Maslow JN. The cost and challenge of vaccine development for emerging and emergent infectious diseases. Lancet Global Health (2018) 6(12):e1266–e7. doi: 10.1016/S2214-109X(18)30418-2 PMC7129672 30342926

[2] Bloom DE Black S Rappuoli R. Emerging infectious diseases: A proactive approach. Proc Natl Acad Sci U.S.A. (2017) 114(16):4055–9. doi: 10.1073/pnas.1701410114 PMC5402424 28396438

[3] Wang C Horby PW Hayden FG Gao GF. A novel coronavirus outbreak of global health concern. Lancet (2020) 395(10223):470–3. doi: 10.1016/S0140-6736(20)30185-9 PMC7135038 31986257

[4] Pronker ES Weenen TC Commandeur H Claassen EHJHM Osterhaus ADME. Risk in vaccine research and development quantified. PLoS One (2013) 8(3):e57755. doi: 10.1371/journal.pone.0057755 23526951 PMC3603987

[5] Thanh Le T Andreadakis Z Kumar A Gómez Román R Tollefsen S Saville M. The covid-19 vaccine development landscape. Nat Rev Drug Discovery (2020) 19(5):305–6. doi: 10.1038/d41573-020-00073-5 32273591

[6] Organisation WH. Who issues its first emergency use validation for a covid-19 vaccine and emphasizes need for equitable global access who.int. In: World health organisation Geneva (2020). Available at: https://www.who.int/news/item/31-12-2020-who-issues-its-first-emergency-use-validation-for-a-covid-19-vaccine-and-emphasizes-need-for-equitable-global-access.

[7] van Doremalen N Lambe T Spencer A Belij-Rammerstorfer S Purushotham JN Port JR. Chadox1 ncov-19 vaccine prevents sars-Cov-2 pneumonia in rhesus macaques. Nature (2020) 586(7830):578–82. doi: 10.1038/s41586-020-2608-y PMC8436420 32731258

[8] Corbett KS Flynn B Foulds KE Francica JR Boyoglu-Barnum S Werner AP. Evaluation of the mrna-1273 vaccine against sars-Cov-2 in nonhuman primates. N Engl J Med (2020) 383(16):1544–55. doi: 10.1056/NEJMoa2024671 PMC7449230 32722908

[9] Watterson D Wijesundara DK Modhiran N Mordant FL Li Z Avumegah MS. Preclinical development of a molecular clamp-stabilised subunit vaccine for severe acute respiratory syndrome coronavirus 2. Clin Transl Immunol (2021) 10(4):e1269–e. doi: 10.1002/cti2.1269 PMC8021130 33841880

[10] Folegatti PM Ewer KJ Aley PK Angus B Becker S Belij-Rammerstorfer S. Safety and immunogenicity of the Chadox1 ncov-19 vaccine against sars-Cov-2: A preliminary report of a phase 1/2, single-blind, randomised controlled trial. Lancet (2020) 396(10249):467–78. doi: 10.1016/s0140-6736(20)31604-4 PMC7445431 32702298

[11] Walsh EE Frenck RW Falsey AR Kitchin N Absalon J Gurtman A. Safety and immunogenicity of two rna-based covid-19 vaccine candidates. New Engl J Med (2020) 383(25):2439–50. doi: 10.1056/NEJMoa2027906 PMC7583697 33053279

[12] Tebas P Yang S Boyer JD Reuschel EL Patel A Christensen-Quick A. Safety and immunogenicity of ino-4800 DNA vaccine against sars-Cov-2: A preliminary report of an open-label, phase 1 clinical trial. EClinicalMedicine (2021) 31:100689. doi: 10.1016/j.eclinm.2020.100689 33392485 PMC7759123

[13] Rey FA Lok SM. Common features of enveloped viruses and implications for immunogen design for next-generation vaccines. Cell (2018) 172(6):1319–34. doi: 10.1016/j.cell.2018.02.054 PMC7112304 29522750

[14] McLellan JS Chen M Joyce MG Sastry M Stewart-Jones GB Yang Y. Structure-based design of a fusion glycoprotein vaccine for respiratory syncytial virus. Science (2013) 342(6158):592–8. doi: 10.1126/science.1243283 PMC4461862 24179220

[15] Corti D Voss J Gamblin SJ Codoni G Macagno A Jarrossay D. A neutralizing antibody selected from plasma cells that binds to group 1 and group 2 influenza a hemagglutinins. Science (2011) 333(6044):850–6. doi: 10.1126/science.1205669 21798894

[16] Pallesen J Wang N Corbett KS Wrapp D Kirchdoerfer RN Turner HL. Immunogenicity and structures of a rationally designed prefusion mers-cov spike antigen. Proc Natl Acad Sci United States America (2017) 114(35):E7348–E57. doi: 10.1073/pnas.1707304114 PMC5584442 28807998

[17] Frey G Chen J Rits-Volloch S Freeman MM Zolla-Pazner S Chen B. Distinct conformational states of hiv-1 Gp41 are recognized by neutralizing and non-neutralizing antibodies. Nat Struct Mol Biol (2010) 17(12):1486–91. doi: 10.1038/nsmb.1950 PMC2997185 21076402

[18] Chan YP Lu M Dutta S Yan L Barr J Flora M. Biochemical, conformational, and immunogenic analysis of soluble trimeric forms of henipavirus fusion glycoproteins. J Virol (2012) 86(21):11457–71. doi: 10.1128/jvi.01318-12 PMC3486283 22915804

[19] Mire CE Chan YP Borisevich V Cross RW Yan L Agans KN. A cross-reactive humanized monoclonal antibody targeting fusion glycoprotein function protects ferrets against lethal nipah virus and hendra virus infection. J Infect Dis (2020) 221(Suppl 4):S471–s9. doi: 10.1093/infdis/jiz515 PMC7199785 31686101

[20] Dang HV Cross RW Borisevich V Bornholdt ZA West BR Chan YP. Broadly neutralizing antibody cocktails targeting nipah virus and hendra virus fusion glycoproteins. Nat Struct Mol Biol (2021) 28(5):426–34. doi: 10.1038/s41594-021-00584-8 PMC12334189 33927387

[21] Graham BS Gilman MSA McLellan JS. Structure-based vaccine antigen design. Annu Rev Med (2019) 70:91–104. doi: 10.1146/annurev-med-121217-094234 30691364 PMC6936610

[22] Moyle PM Toth I. Modern subunit vaccines: Development, components, and research opportunities. ChemMedChem (2013) 8(3):360–76. doi: 10.1002/cmdc.201200487 23316023

[23] Pancera M Zhou T Druz A Georgiev IS Soto C Gorman J. Structure and immune recognition of trimeric pre-fusion hiv-1 env. Nature (2014) 514(7523):455–61. doi: 10.1038/nature13808 PMC4348022 25296255

[24] Wijesundara DK Avumegah MS Lackenby J Modhiran N Isaacs A Young PR. Rapid response subunit vaccine design in the absence of structural information. Front Immunol (2020) 11:592370. doi: 10.3389/fimmu.2020.592370 33250897 PMC7672035

[25] McMillan CLD Cheung STM Modhiran N Barnes J Amarilla AA Bielefeldt-Ohmann H. Development of molecular clamp stabilized hemagglutinin vaccines for influenza a viruses. NPJ Vaccines (2021) 6(1):135. doi: 10.1038/s41541-021-00395-4 34750396 PMC8575991

[26] Chappell KJ Mordant FL Li Z Wijesundara DK Ellenberg P Lackenby JA. Safety and immunogenicity of an Mf59-adjuvanted spike glycoprotein-clamp vaccine for sars-Cov-2: A randomised, double-blind, placebo-controlled, phase 1 trial. Lancet Infect Dis (2021) 21(10):1383–94. doi: 10.1016/S1473-3099(21)00200-0 PMC8055208 33887208

[27] Hastie KM Zandonatti MA Kleinfelter LM Heinrich ML Rowland MM Chandran K. Structural basis for antibody-mediated neutralization of lassa virus. Sci (New York NY) (2017) 356(6341):923–8. doi: 10.1126/science.aam7260 PMC6007842 28572385

[28] Li Y Wan Y Liu P Zhao J Lu G Qi J. A humanized neutralizing antibody against mers-cov targeting the receptor-binding domain of the spike protein. Cell Res (2015) 25(11):1237–49. doi: 10.1038/cr.2015.113 PMC4650419 26391698

[29] Ying T Prabakaran P Du L Shi W Feng Y Wang Y. Junctional and allele-specific residues are critical for mers-cov neutralization by an exceptionally potent germline-like antibody. Nat Commun (2015) 6:8223. doi: 10.1038/ncomms9223 26370782 PMC4571279

[30] Walls AC Xiong X Park YJ Tortorici MA Snijder J Quispe J. Unexpected receptor functional mimicry elucidates activation of coronavirus fusion. Cell (2019) 176(5):1026–39.e15. doi: 10.1016/j.cell.2018.12.028 30712865 PMC6751136

[31] Yu X Zhang S Jiang L Cui Y Li D Wang D. Structural basis for the neutralization of mers-cov by a human monoclonal antibody mers-27. Sci Rep (2015) 5:13133. doi: 10.1038/srep13133 26281793 PMC4539535

[32] Chen Z Bao L Chen C Zou T Xue Y Li F. Human neutralizing monoclonal antibody inhibition of middle East respiratory syndrome coronavirus replication in the common marmoset. J Infect Dis (2017) 215(12):1807–15. doi: 10.1093/infdis/jix209 PMC7107363 28472421

[33] Wang L Shi W Chappell JD Joyce MG Zhang Y Kanekiyo M. Importance of neutralizing monoclonal antibodies targeting multiple antigenic sites on the middle East respiratory syndrome coronavirus spike glycoprotein to avoid neutralization escape. J Virol (2018) 92(10):e02002–17. doi: 10.1128/jvi.02002-17 PMC5923077 29514901

[34] Wang L Shi W Joyce MG Modjarrad K Zhang Y Leung K. Evaluation of candidate vaccine approaches for mers-cov. Nat Commun (2015) 6(1):7712. doi: 10.1038/ncomms8712 26218507 PMC4525294

[35] Robinson JE Hastie KM Cross RW Yenni RE Elliott DH Rouelle JA. Most neutralizing human monoclonal antibodies target novel epitopes requiring both lassa virus glycoprotein subunits. Nat Commun (2016) 7:11544. doi: 10.1038/ncomms11544 27161536 PMC4866400

[36] Bornholdt ZA Turner HL Murin CD Li W Sok D Souders CA. Isolation of potent neutralizing antibodies from a survivor of the 2014 Ebola virus outbreak. Science (2016) 351(6277):1078–83. doi: 10.1126/science.aad5788 PMC4900763 26912366

[37] Maruyama T Rodriguez LL Jahrling PB Sanchez A Khan AS Nichol ST. Ebola Virus can be effectively neutralized by antibody produced in natural human infection. J Virol (1999) 73(7):6024–30. doi: 10.1128/JVI.73.7.6024-6030.1999 PMC112663 10364354

[38] Qiu X Alimonti JB Melito PL Fernando L Ströher U Jones SM. Characterization of Zaire ebolavirus glycoprotein-specific monoclonal antibodies. Clin Immunol (2011) 141(2):218–27. doi: 10.1016/j.clim.2011.08.008 21925951

[39] Corti D Misasi J Mulangu S Stanley DA Kanekiyo M Wollen S. Protective monotherapy against lethal Ebola virus infection by a potently neutralizing antibody. Science (2016) 351(6279):1339–42. doi: 10.1126/science.aad5224 26917593

[40] Wilson JA Hevey M Bakken R Guest S Bray M Schmaljohn AL. Epitopes involved in antibody-mediated protection from Ebola virus. Science (2000) 287(5458):1664–6. doi: 10.1126/science.287.5458.1664 10698744

[41] Avanzato VA Oguntuyo KY Escalera-Zamudio M Gutierrez B Golden M Kosakovsky Pond SL. A structural basis for antibody-mediated neutralization of nipah virus reveals a site of vulnerability at the fusion glycoprotein apex. Proc Natl Acad Sci U.S.A. (2019) 116(50):25057–67. doi: 10.1073/pnas.1912503116 PMC6911215 31767754

[42] Dang HV Chan YP Park YJ Snijder J Da Silva SC Vu B. An antibody against the f glycoprotein inhibits nipah and hendra virus infections. Nat Struct Mol Biol (2019) 26(10):980–7. doi: 10.1038/s41594-019-0308-9 PMC6858553 31570878

[43] Marzi A Chadinah S Haddock E Feldmann F Arndt N Martellaro C. Recently identified mutations in the Ebola virus-makona genome do not alter pathogenicity in animal models. Cell Rep (2018) 23(6):1806–16. doi: 10.1016/j.celrep.2018.04.027 PMC5969531 29742435

[44] Reed LJ Muench H. A simple method of estimating fifty per cent endpoints. Am J Epidemiol (1938) 27(3):493–7. doi: 10.1093/oxfordjournals.aje.a118408

[45] Li K Wohlford-Lenane CL Channappanavar R Park J-E Earnest JT Bair TB. Mouse-adapted mers coronavirus causes lethal lung disease in human Dpp4 knockin mice. Proc Natl Acad Sci (2017) 114(15):E3119–E28. doi: 10.1073/pnas.1619109114 PMC5393213 28348219

[46] Li K Wohlford-Lenane C Perlman S Zhao J Jewell AK Reznikov LR. Middle East respiratory syndrome coronavirus causes multiple organ damage and lethal disease in mice transgenic for human dipeptidyl peptidase 4. J Infect Dis (2016) 213(5):712–22. doi: 10.1093/infdis/jiv499 PMC4747621 26486634

[47] Bray M Davis K Geisbert T Schmaljohn C Huggins J. A mouse model for evaluation of prophylaxis and therapy of Ebola hemorrhagic fever. J Infect Dis (1998) 178(3):651–61. doi: 10.1086/515386 9728532

[48] Marzi A Robertson SJ Haddock E Feldmann F Hanley PW Scott DP. Vsv-ebov rapidly protects macaques against infection with the 2014/15 Ebola virus outbreak strain. Science (2015) 349(6249):739–42. doi: 10.1126/science.aab3920 PMC11040598 26249231

[49] Baer A Kehn-Hall K. Viral concentration determination through plaque assays: Using traditional and novel overlay systems. J Vis Exp (2014) 93):e52065. doi: 10.3791/52065 PMC4255882 25407402

[50] Pyankov OV Setoh YX Bodnev SA Edmonds JH Pyankova OG Pyankov SA. Successful post-exposure prophylaxis of Ebola infected non-human primates using Ebola glycoprotein-specific equine igg. Sci Rep (2017) 7:41537. doi: 10.1038/srep41537 28155869 PMC5290740

[51] Thakur N Gallo G Elreafey AME Bailey D. Production of recombinant replication-defective lentiviruses bearing the sars-cov or sars-Cov-2 attachment spike glycoprotein and their application in receptor tropism and neutralisation assays. Bio Protoc (2021) 11(21):e4249. doi: 10.21769/BioProtoc.4249 PMC8595443 34859135

[52] Thakur N Conceicao C Isaacs A Human S Modhiran N McLean RK. Micro-fusion inhibition tests: Quantifying antibody neutralization of virus-mediated cell-cell fusion. J Gen Virol (2021) 102(1):jgv001506. doi: 10.1099/jgv.0.001506 PMC8116787 33054904

[53] Isaacs A Cheung STM Thakur N Jaberolansar N Young A Modhiran N. Combinatorial f-G immunogens as nipah and respiratory syncytial virus vaccine candidates. Viruses (2021) 13(10):1942. doi: 10.3390/v13101942 34696372 PMC8537613

[54] Organisation WH. Prioritizing diseases for research and development in emergency contexts who.int. In: World health organisation. Geneva (2020). Available at: https://www.who.int/activities/prioritizing-diseases-for-research-and-development-in-emergency-contexts#:~:text=At%20present%2C%20the%20priority%20diseases,disease%20and%20Marburg%20virus%20disease.

[55] Chappell KJ Watterson D Young PR. Chimeric molecules and uses thereof. (International Bureau: World Intellectual Property Organization) (2018). Patent number: WO2018176103A1. Available at: https://patents.google.com/patent/WO2018176103A1/en

[56] Loomis RJ Stewart-Jones GBE Tsybovsky Y Caringal RT Morabito KM McLellan JS. Structure-based design of nipah virus vaccines: A generalizable approach to paramyxovirus immunogen development. Front Immunol (2020) 11:842. doi: 10.3389/fimmu.2020.00842 32595632 PMC7300195

[57] Tsuda Y Safronetz D Brown K LaCasse R Marzi A Ebihara H. Protective efficacy of a bivalent recombinant vesicular stomatitis virus vaccine in the Syrian hamster model of lethal Ebola virus infection. J Infect Dis (2011) 204 Suppl 3(Suppl 3):S1090–S7. doi: 10.1093/infdis/jir379 PMC3189997 21987746

[58] Normile D. Development of unique Australian covid-19 vaccine halted. Science (2020), 80–2020. doi: 10.1126/science.abg1208

[59] Evan GI Lewis GK Ramsay G Bishop JM. Isolation of monoclonal antibodies specific for human c-myc proto-oncogene product. Mol Cell Biol (1985) 5(12):3610–6. doi: 10.1128/mcb.5.12.3610-3616.1985 PMC369192 3915782

[60] Hopp TP Prickett KS Price VL Libby RT March CJ Pat Cerretti D. A short polypeptide marker sequence useful for recombinant protein identification and purification. Bio/Technology (1988) 6(10):1204–10. doi: 10.1038/nbt1088-1204

[61] Moser AC Hage DS. Immunoaffinity chromatography: An introduction to applications and recent developments. Bioanalysis (2010) 2(4):769–90. doi: 10.4155/bio.10.31 PMC2903764 20640220

[62] Cromwell ME Hilario E Jacobson F. Protein aggregation and bioprocessing. AAPS J (2006) 8(3):E572–9. doi: 10.1208/aapsj080366 PMC2761064 17025275

[63] Pelliccia M Andreozzi P Paulose J D’Alicarnasso M Cagno V Donalisio M. Additives for vaccine storage to improve thermal stability of adenoviruses from hours to months. Nat Commun (2016) 7(1):13520. doi: 10.1038/ncomms13520 27901019 PMC5141364

[64] Pollard AJ Bijker EM. A guide to vaccinology: From basic principles to new developments. Nat Rev Immunol (2021) 21(2):83–100. doi: 10.1038/s41577-020-00479-7 33353987 PMC7754704

[65] Hastie KM Saphire EO. Lassa virus glycoprotein: Stopping a moving target. Curr Opin Virol (2018) 31:52–8. doi: 10.1016/j.coviro.2018.05.002 PMC6193841 29843991

[66] Sommerstein R Flatz L Remy MM Malinge P Magistrelli G Fischer N. Arenavirus glycan shield promotes neutralizing antibody evasion and protracted infection. PLoS Pathog (2015) 11(11):e1005276. doi: 10.1371/journal.ppat.1005276 26587982 PMC4654586

[67] Salami K Gouglas D Schmaljohn C Saville M Tornieporth N. A review of lassa fever vaccine candidates. Curr Opin Virol (2019) 37:105–11. doi: 10.1016/j.coviro.2019.07.006 31472333

[68] Cai Y Ye C Cheng B Nogales A Iwasaki M Yu S. A lassa fever live-attenuated vaccine based on codon deoptimization of the viral glycoprotein gene. mBio (2020) 11(1):e00039–20. doi: 10.1128/mBio.00039-20 PMC7042690 32098811

[69] Abreu-Mota T Hagen KR Cooper K Jahrling PB Tan G Wirblich C. Non-neutralizing antibodies elicited by recombinant lassa-rabies vaccine are critical for protection against lassa fever. Nat Commun (2018) 9(1):4223. doi: 10.1038/s41467-018-06741-w 30310067 PMC6181965

[70] Lee JE Saphire EO. Neutralizing ebolavirus: Structural insights into the envelope glycoprotein and antibodies targeted against it. Curr Opin Struct Biol (2009) 19(4):408–17. doi: 10.1016/j.sbi.2009.05.004 PMC2759674 19559599

[71] Misasi J Gilman MS Kanekiyo M Gui M Cagigi A Mulangu S. Structural and molecular basis for Ebola virus neutralization by protective human antibodies. Science (2016) 351(6279):1343–6. doi: 10.1126/science.aad6117 PMC5241105 26917592

[72] Sullivan NJ Martin JE Graham BS Nabel GJ. Correlates of protective immunity for Ebola vaccines: Implications for regulatory approval by the animal rule. Nat Rev Microbiol (2009) 7(5):393–400. doi: 10.1038/nrmicro2129 19369954 PMC7097244

[73] Isaacs A Li Z Cheung STM Wijesundara DK McMillan CLD Modhiran N. Adjuvant selection for influenza and rsv prefusion subunit vaccines. Vaccines (Basel) (2021) 9(2):71. doi: 10.3390/vaccines9020071 33498370 PMC7909420

[74] Extance A. Mrna vaccines: Hope beneath the hype thebmj (2021). Available at: https://www.bmj.com/content/375/bmj.n2744. 10.1136/bmj.n2744 34819269

[75] Crommelin DJA Anchordoquy TJ Volkin DB Jiskoot W Mastrobattista E. Addressing the cold reality of mrna vaccine stability. J Pharm Sci (2021) 110(3):997–1001. doi: 10.1016/j.xphs.2020.12.006 33321139 PMC7834447

[76] Jackson LA Anderson EJ Rouphael NG Roberts PC Makhene M Coler RN. An mrna vaccine against sars-Cov-2 - preliminary report. N Engl J Med (2020) 383(20):1920–31. doi: 10.1056/NEJMoa2022483 PMC7377258 32663912

[77] Vogel AB Kanevsky I Che Y Swanson KA Muik A Vormehr M. BNT162b vaccines protect rhesus macaques from SARS-CoV-2. Nature (2021) 592:283–89. doi: 10.1038/s41586-021-03275 33524990

