# Supplementary material for: A platform technology for generating subunit vaccines against diverse viral pathogens
Source: Front Immunol. 2022 Aug 18;13:963023. doi: 10.3389/fimmu.2022.963023 (PMC9436389; doi:10.3389/fimmu.2022.963023)
Supplement: Supplementary file 1 [file DataSheet_1.docx]

Supplementary Material

*A Platform Technology for Generating Subunit Vaccines Against Diverse Viral Pathogens*

## Supplementary Figures

##
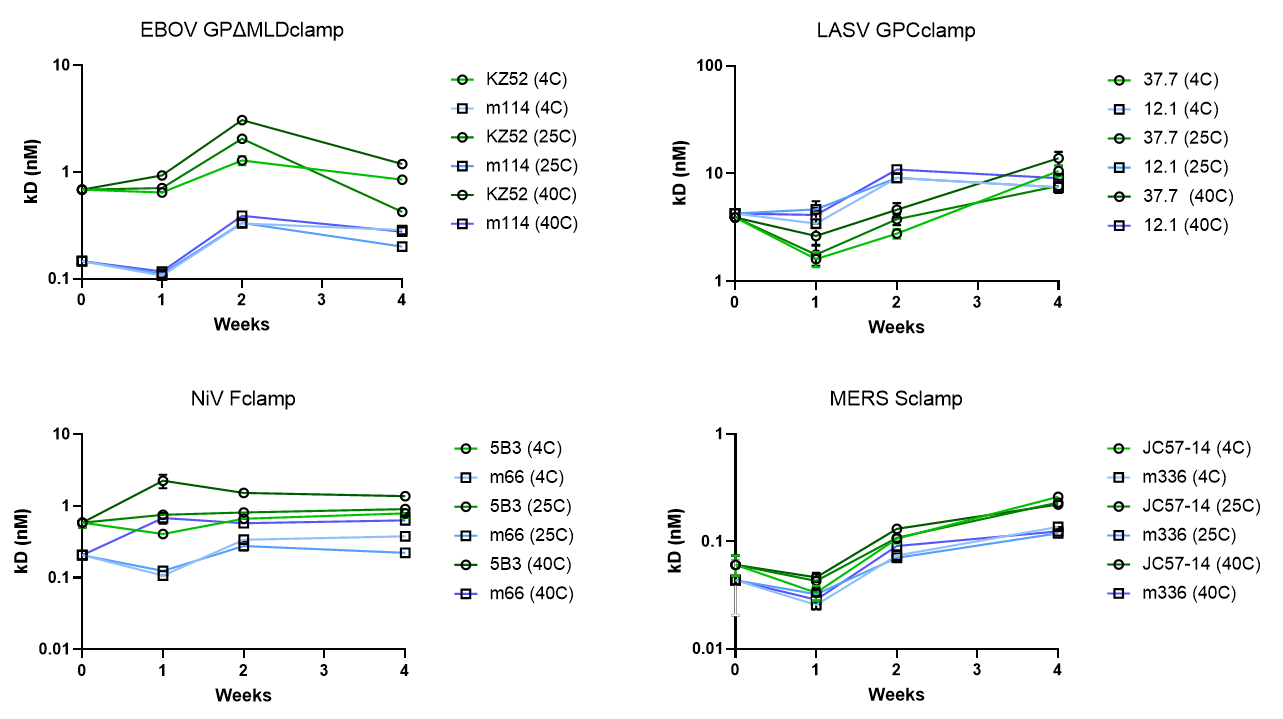


**Figure S1.** **Thermostability of clamped vaccine antigens.** Replicate aliquots of each of the clamped antigens were incubated at 4 °C, 25 °C and 40 °C. Antigens were then assayed by ELISA at 0, 1, 2 and 4 weeks using the stated antibodies. Dissociation constants (kD) were calculated using a one-site specific binding model in GraphPad Prism.


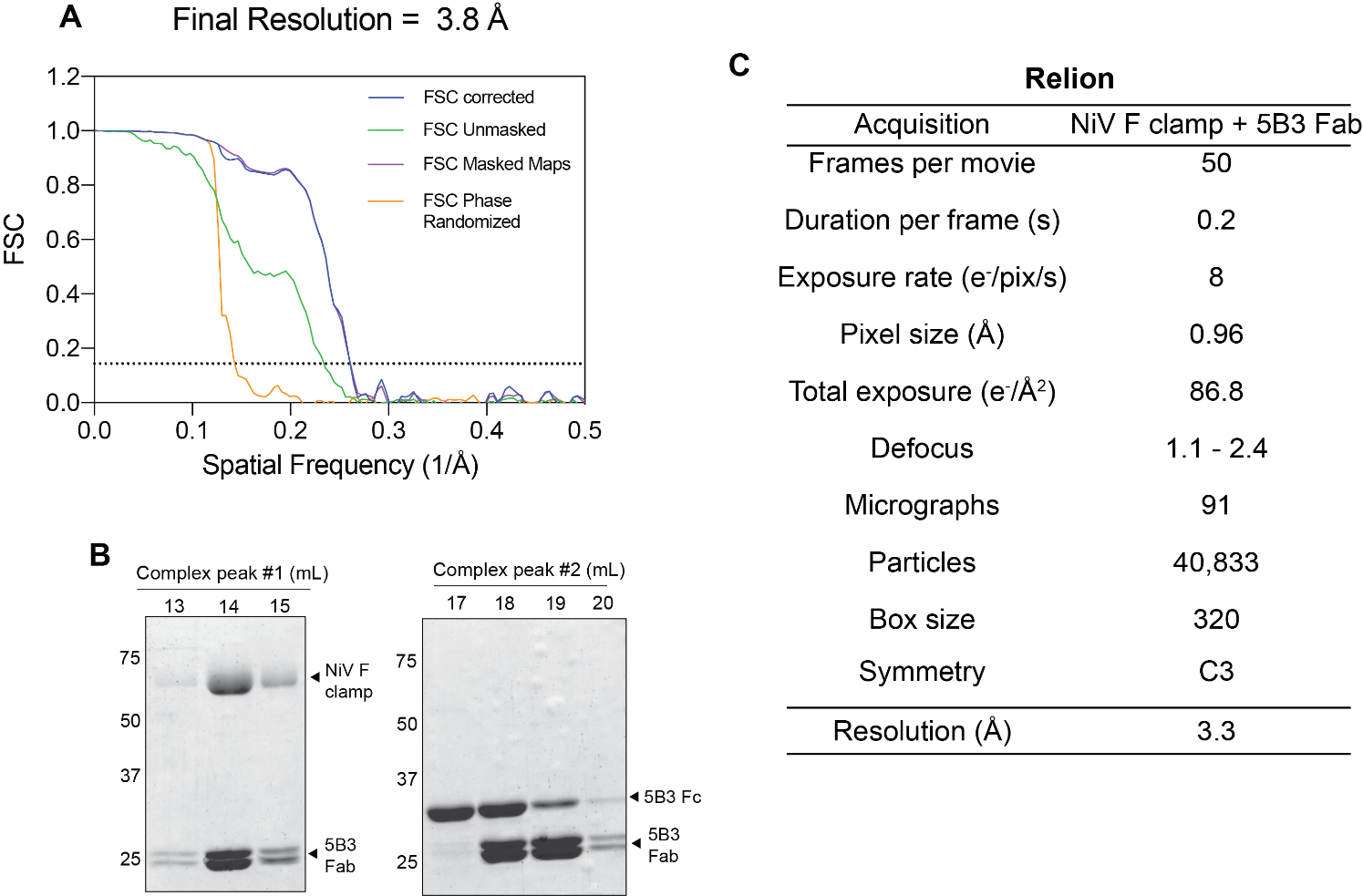


**Figure S2. Cryo-EM acquisition details.** (A) Fourier shell correlation (FSC) of NiV F 5B3-complexed data set. (B) Coomassie-stained SDS-PAGE of SEC NiV F clamp 5B3 complexed peaks. (C) Cryo-EM scope acquisition details.


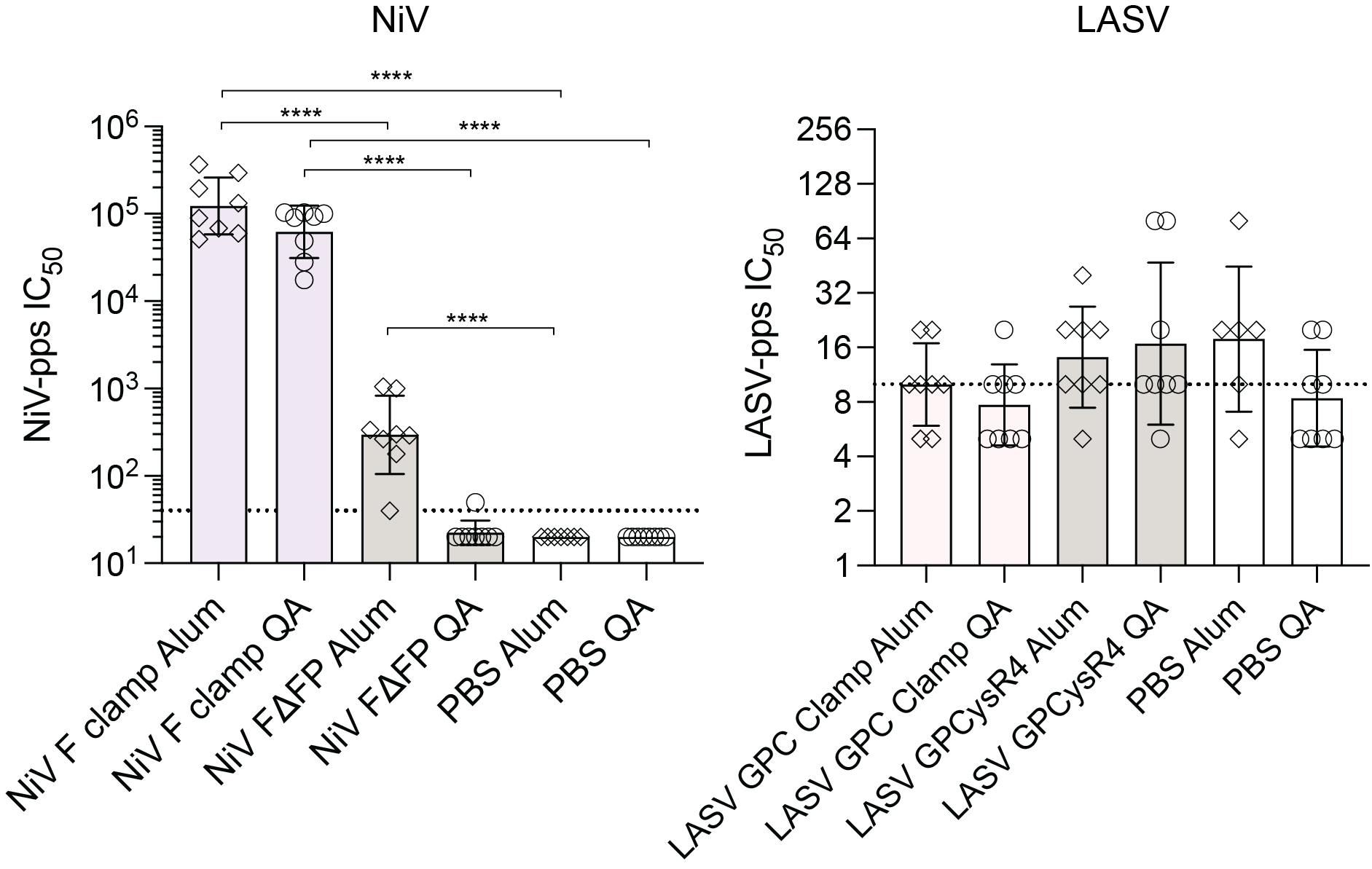


**Figure S3. Pseudovirus neutralisation of NiV and LASV.** Mouse serum samples were assessed for neutralisation against lentivirus pseudoviruses (pps), with reciprocal inhibitory concentrations 50% (IC50) shown. Dotted line shows assay limit of detection. Statistics calculated using one-way ANOVA with Tukey’s correction.

**Table S1. Dissociation constants of prefusion-specific monoclonal antibodies against the recombinant antigens.** Monoclonal antibodies were titrated against the recombinant antigens by ELISA. Dissociation constants (kD) were calculated using a one-site-specific binding model using GraphPad Prism.

*NB: no binding

| **Viral antigen** | **mAb** | **K_d_ nM (SEM) – clamp stabilised antigen** | **K_d_ nM (SEM) –unstabilised antigen** |
| --- | --- | --- | --- |
| EBOV GGPGP∆MLD | h15758 | 2.88 (0.33) | 2.16 (0.11) |
|  | h15765 | 0.47 (0.05) | 0.79 (0.11) |
|  | h15959 | 1.63 (0.24) | 2.11 (0.15) |
|  | h15960 | 5.1 (0.51) | 4.4 (0.41) |
|  | hKZ52 | 1.83 (0.15) | 1.7 (0.17) |
|  | h2G4 | 1.88 (0.17) | 1.48 (0.2) |
|  | hmAb100 | 0.89 (0.27) | 1.37 (0.34) |
|  | hmAb114 | 0.43 (0.06) | 0.43 (0.05) |
|  | h13C6 | 0.98 (0.11) | 0.21 (0.21) |
|  | h1H3 | 3.76 (0.38) | 5.72 (0.63) |
|  | h16042 | 0.82 (0.17) | 1.49 (0.16) |
| Nipah F | 5B3 | 0.1 (0.057) | NB |
|  | mAb66 | 0.13 (0.03) | NB |
| MERS S | 4C2 | 0.13 (0.006) | 0.13 (0.007) |
|  | m336 | 0.07 (0.005) | 0.07 (0.003) |
|  | JC57-14 | 24.8 (0.889) | 23.8 (1.58) |
|  | D12 | 24.8 (1.15) | 20.07 (0.91) |
|  | LCA60 | 0.04 (0.005) | 0.04 (0.007) |
|  | MCA1 | 0.04 (0.006) | 0.04 (0.003) |
|  | CDC2-C2 | 0.04 (0.003) | 0.03 (0.003) |
|  | MERS27 | 9.47 (1.26) | 2.9 (0.25) |
|  | G4 | 0.35 (0.026) | 39.3 (5.9) |
| LASV GPC | 37.7H | 0.62 (0.06) | 0.26 (0.03) |
|  | 25.10C | 0.68 (0.3) | 0.38 (0.06) |
|  | 12.1F | 0.77 (0.07) | 0.27 (0.01) |
